# Supplementary material for: An epigenetic predictor of death captures multi-modal measures of brain health
Source: Mol Psychiatry. 2019 Dec 3;26(8):3806–16. doi: 10.1038/s41380-019-0616-9 (PMC8550950; doi:10.1038/s41380-019-0616-9)
Supplement: Supplementary file 2 — Supplementary Figures 1-7 [file 41380_2019_616_MOESM2_ESM.pdf]

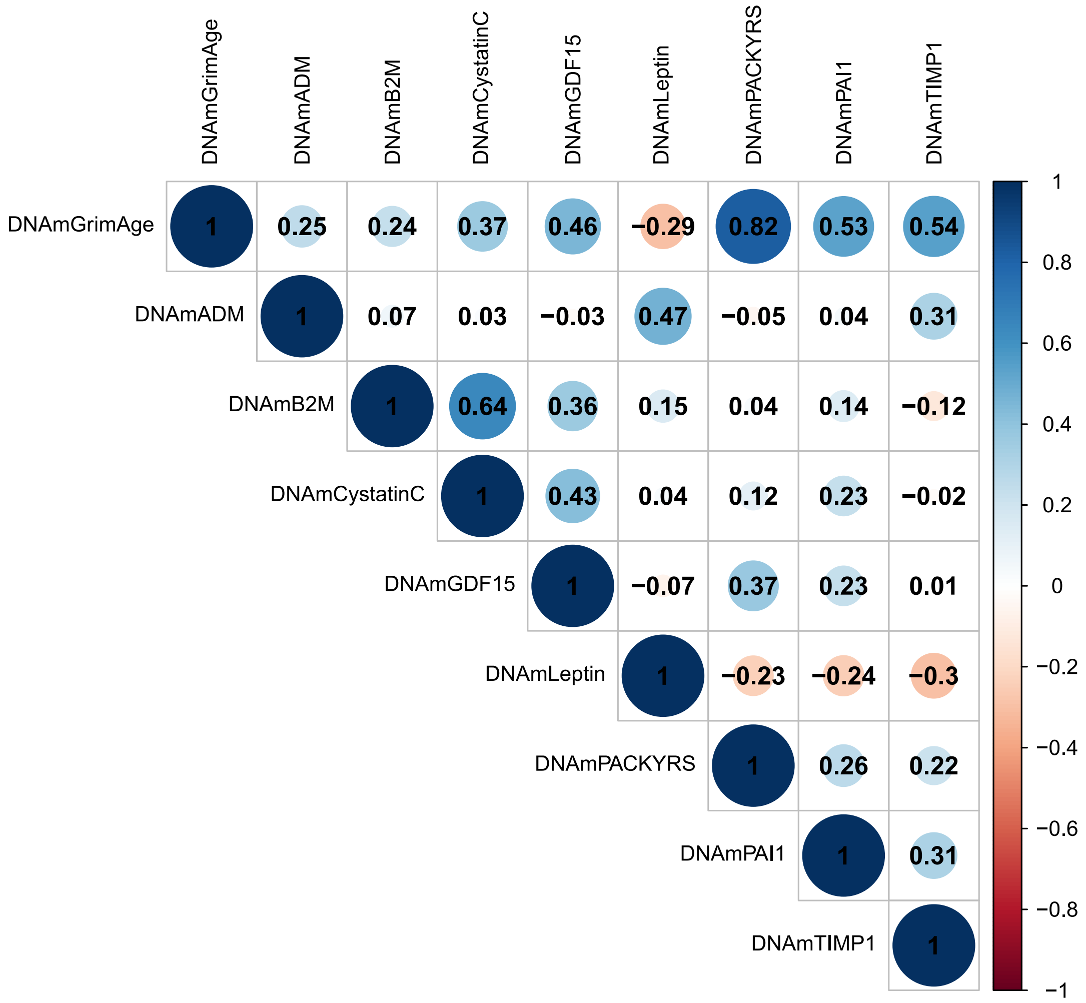

Supplementary Figure 1. Correlation between DNAm GrimAge and the individual components which comprise the clock. This figure demonstrates the degree of correlation amongst individual components whose linear combination gives rise to measures of DNAm GrimAge within the LBC1936 study.

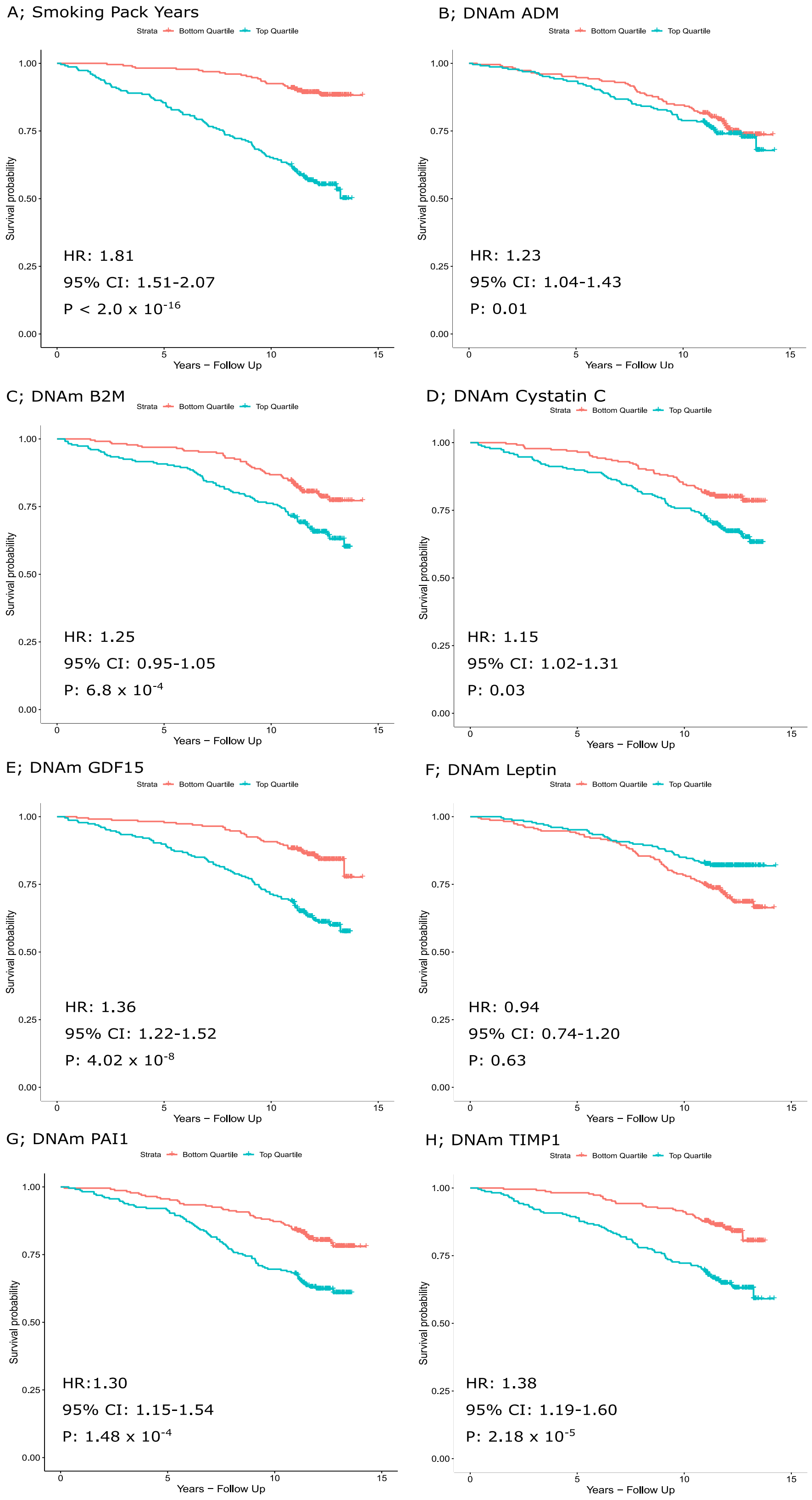

Supplementary Figure 2. Cox proportional hazard models for individual components of DNAm GrimAge. Individual panels show difference in survival probabilities between top and bottom quartiles of DNAm GrimAge components. Output from Cox proportional hazard models are also displayed in the relevant panels.

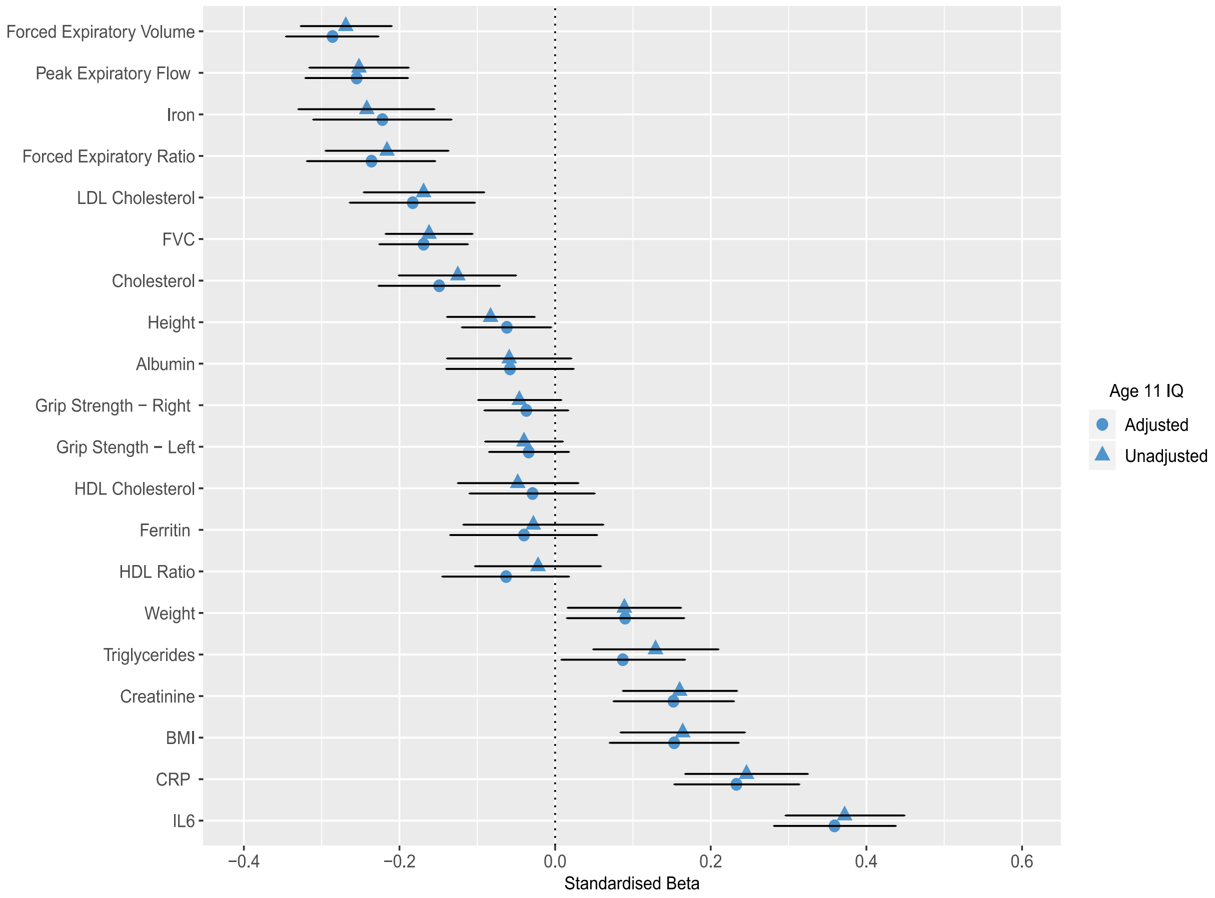

Supplementary Figure 3. The association between age-adjusted DNAm GrimAge and physical and blood traits in the LBC1936 study. The outputs from regression models examining the association between age-adjusted DNAm GrimAge and physical and blood traits are shown. Models adjusted and unadjusted for age 11 IQ are represented by circles and triangles, respectively. BMI (body mass index), CRP (C-reactive protein), FVC (forced vital capacity), HDL (high-density lipoprotein), IL6 (interleukin 6).

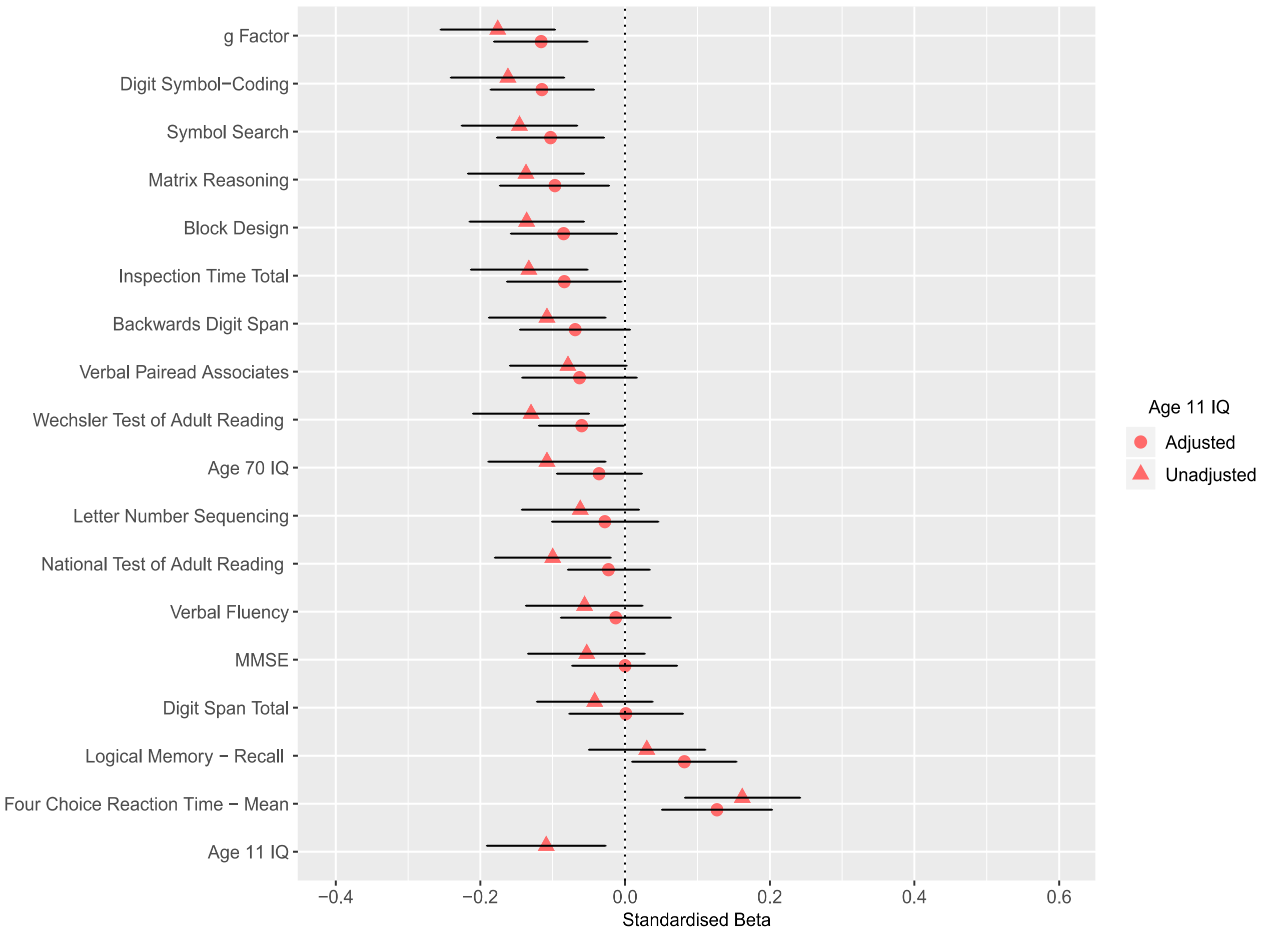

Supplementary Figure 4. The association between age-adjusted DNAm GrimAge and cognitive traits in the LBC1936 study. The outputs from regression models examining the association between age-adjusted DNAm GrimAge and cognitive traits are shown. Models adjusted and unadjusted for age 11 IQ are represented by circles and triangles, respectively. MMSE (mini-mental state examination).

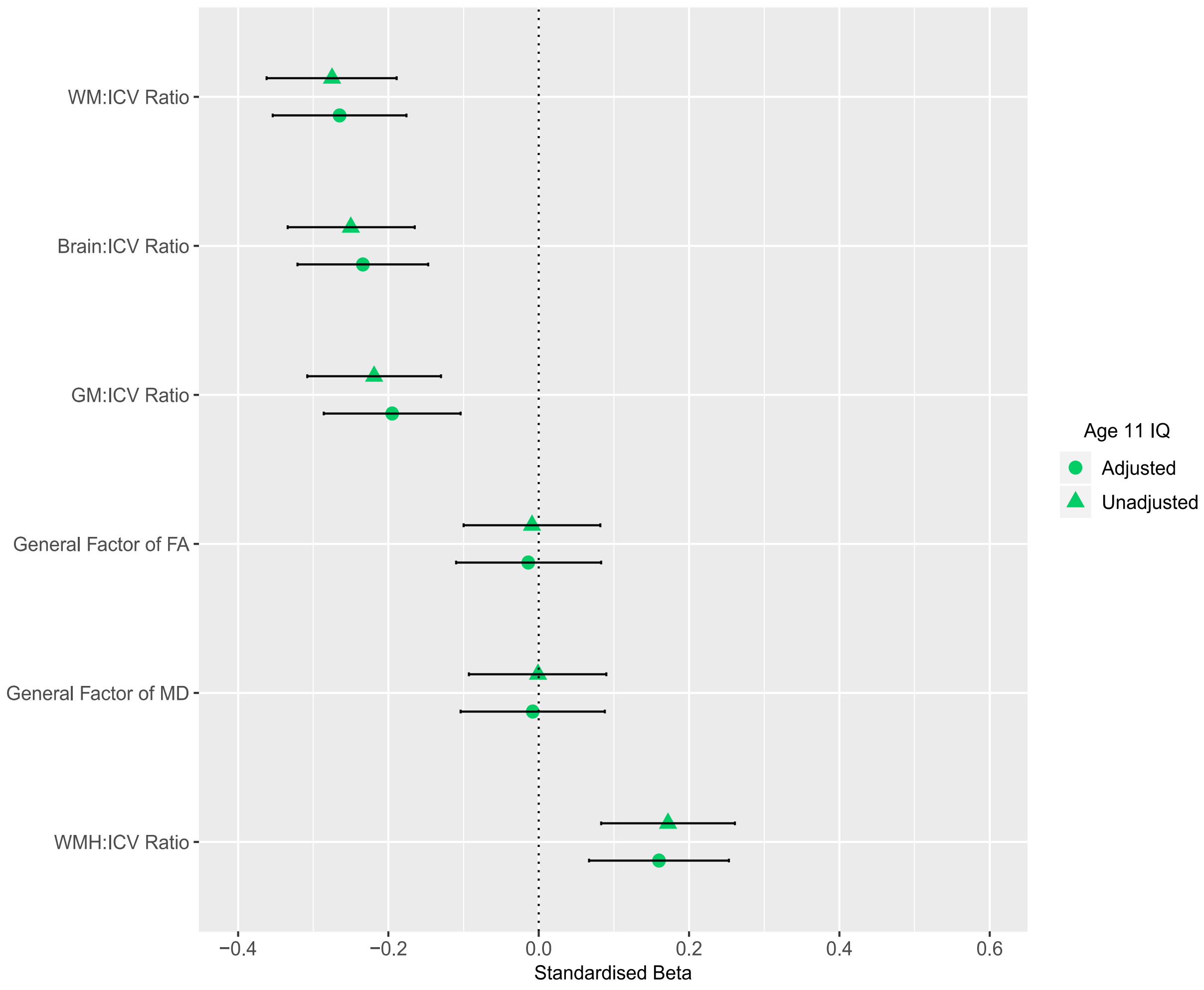

Supplementary Figure 5. The association between age-adjusted DNAm GrimAge and neuroimaging traits in the LBC1936 study. The outputs from regression models examining the association between age-adjusted DNAm GrimAge and neuroimaging traits are shown. Models adjusted and unadjusted for age 11 IQ are represented by circles and triangles, respectively. FA (fractional anisotropy), GM (grey matter), ICV (intracranial volume), MD (mean diffusivity), WM (white matter), WMH (white matter hyperintensities).

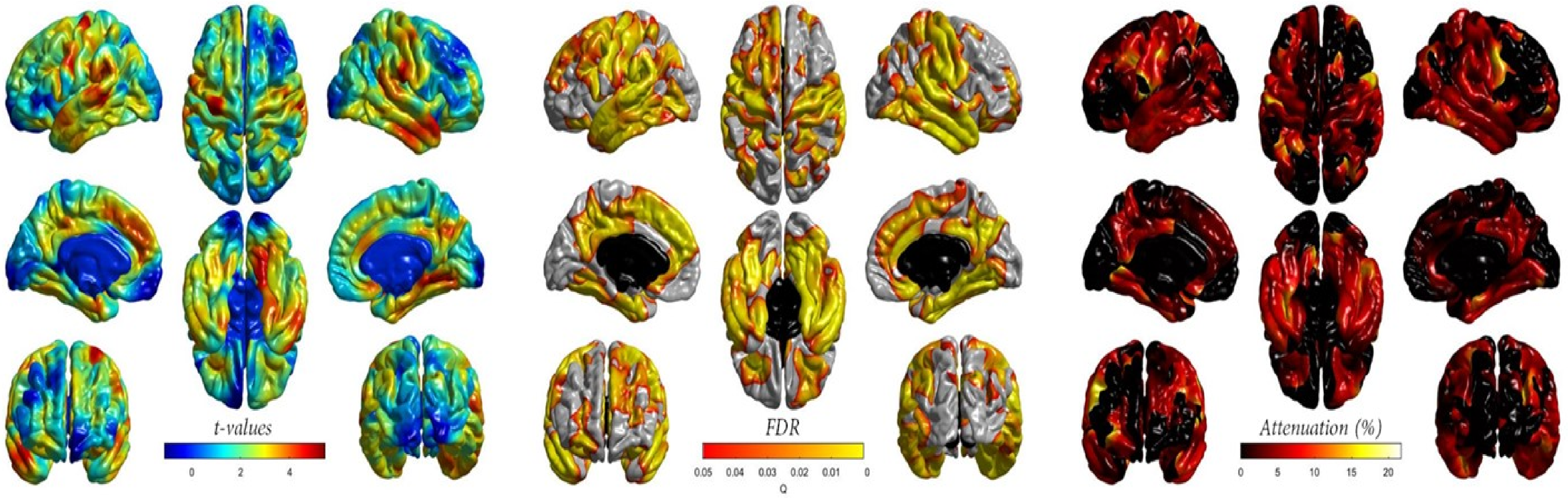

Supplementary Figure 6. Cross-sectional association between age-adjusted DNAm GrimAge and regional cortical volume in the LBC1936, corrected for age 11 IQ. *Left panel:* t-values indicate the magnitude of the negative association (values have been flipped for visualisation purposes). *Centre Panel*: Corresponding FDR-corrected P values indicate the spatial distribution of significant associations. *Right Panel:* Degree of attenuation of t-values at initially FDR-significant loci before and after correcting cortical volumetric-DNAm GrimAge associations for age 11 IQ. FDR (false discovery rate).

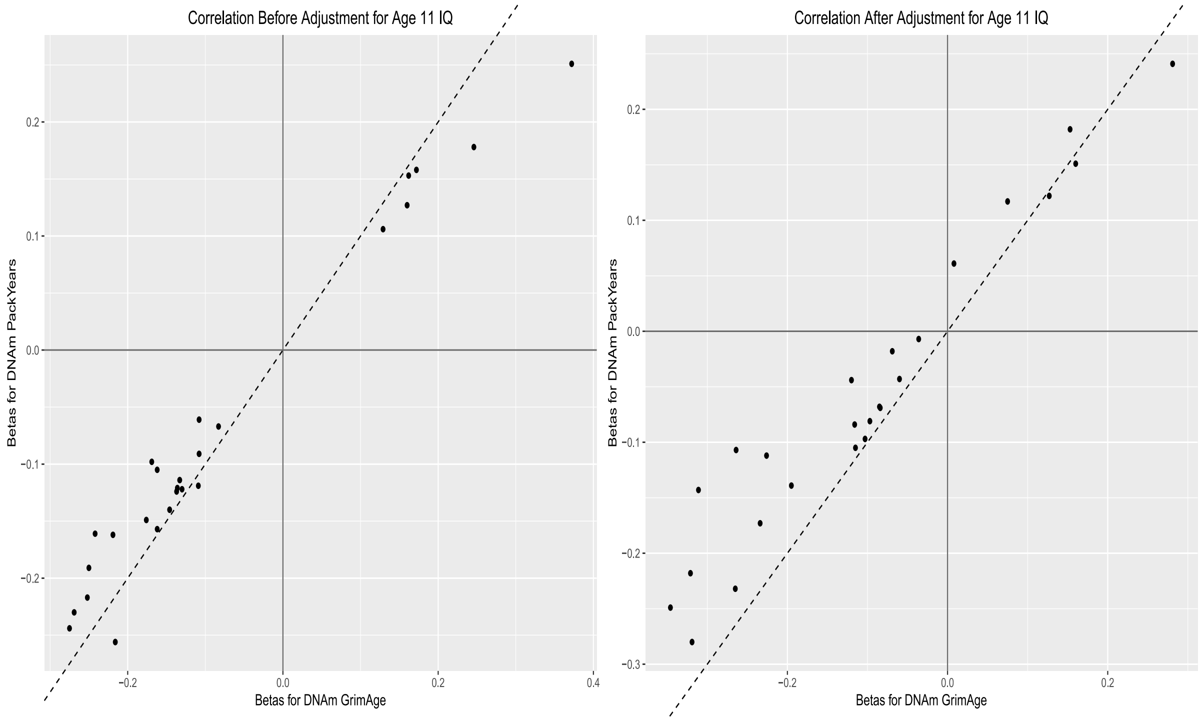

Supplementary Figure 7. The correlation between betas obtained from regression models examining phenotypic associations with DNAm GrimAge and DNAm PackYears, before and after adjustment for age 11 IQ. *Left panel:* Shows the degree of correlation between DNAm GrimAge and DNAm PackYears with phenotypic traits in the LBC1936 study without adjustment for childhood intelligence. *Right panel:* Shows the degree of correlation between phenotypic associations with DNAm GrimAge and DNAm PackYears after adjusting for age 11 IQ.
