# Supplementary material for: An epigenetic predictor of death captures multi-modal measures of brain health
Source: Mol Psychiatry. 2019 Dec 3;26(8):3806–16. doi: 10.1038/s41380-019-0616-9 (PMC8550950; doi:10.1038/s41380-019-0616-9)
Supplement: Supplementary file 4 — Supplementary Note 2 [file 41380_2019_616_MOESM4_ESM.pdf]

Supplementary Note 2. The association of age-adjusted DNAm Pack Years and DNAm blood proteins with physical, cognitive, neuroimaging and protein traits in the LBC1936 study.

DNAm Pack Years

Physical

In relation to physical and blood traits, age-adjusted DNAm Pack Years was positively associated with levels of interleukin-6 (β = 0.25, SE = 0.04, P = 1.6 x 10^{-9}), C-reactive protein (β = 0.18, SE = 0.04, P = 4.3 x 10^{-5}), creatinine (β = 0.13, SE = 0.04, P = 2.0 x 10^{-3}) and triglycerides (β = 0.11, SE = 0.04, P = 0.02). As with DNAm GrimAge, only the association with triglyceride concentration failed to remain significant after adjusting for age 11 IQ (% attenuation: 42.08%).

Age-adjusted DNAm Pack Years was negatively associated with all four measures of lung function (β = [-0.11 to -0.23] SE = [0.03 to 0.04], P = [1.0 x 10^{-3} to 2.0 x 10^{-13}]), levels of iron (β = -0.16, SE = 0.04, P = 1.0 x 10^{-3}), low-density lipoprotein cholesterol (β = -0.10, SE = 0.04, P = 0.03), and height (β = -0.07, SE = 0.03, P = 0.04). Only the association with height failed to remain significant following controlling for childhood cognitive ability (% attenuation 34.33%).

Cognitive

Following adjustment for age 11 IQ, age-adjusted DNAm Pack Years was significantly associated with the g factor (β = -0.08, SE = 0.03, P = 0.03), lower scores on the digit symbol coding (β = -0.11, SE = 0.04, P = 0.01) and symbol search tasks (β = -0.10, SE = 0.04, P = 0.02) and an increased mean four choice reaction time (β = 0.12, SE = 0.04, P = 6.0 x 10^{-3}).

Neuroimaging

Age-adjusted DNAm Pack Years was negatively associated with ratios of white matter volume:ICV (β = -0.25, SE = 0.04, P = 3.0 x 10^{-7}), brain volume:ICV (β = -0.19, SE = 0.04, P = 6.0 x 10^{-5}), grey matter volume:ICV (β = -0.16, SE = 0.04, P = 1.0 x 10^{-3}). Furthermore, age-adjusted DNAm Pack Years was positively associated with the ratio of white matter hyperintensities:ICV (β = 0.16, SE = 0.04, P = 2.0 x 10^{-3}). All associations remained significant after adjusting for age 11 IQ.

Protein

DNAm Pack Years was significantly associated with the levels of 30/92 Olink® proteins, 24 of these associations remained significant following adjustment for age 11 IQ (Supplementary File 5a).

DNAm Adrenomedullin

Physical

In relation to physical and blood traits, age-adjusted DNAm Adrenomedullin (ADM) was positively associated with levels of interleukin-6 (β = 0.30, SE = 0.04, P = 8.6 x 10^{-10}), C-reactive protein (β = 0.15, SE = 0.04, P = 0.01), higher body mass index (β = 0.21, SE = 0.04, P = 1.7 x 10^{-4}) and weight (β = 0.13, SE = 0.04, P = 0.01). All associations remained significant after adjusting for age 11 IQ.

Age-adjusted DNAm ADM was negatively associated with three measures of lung function: peak expiratory flow (β = -0.12, SE = 0.04, P = 0.01), forced expiratory volume (β = -0.10, SE = 0.03, P = 0.02), forced vital capacity (β = -0.10, SE = 0.03, P = 0.02) and levels of iron (β = -0.16, SE = 0.05, P = 0.01). All associations remained significant after adjusting for age 11 IQ.

Cognitive

Following adjustment for age 11 IQ, age-adjusted ADM was significantly associated with the g factor (β = -0.10, SE = 0.04, P = 0.03).

Neuroimaging

Age-adjusted DNAm ADM was not significantly associated with any neuroimaging phenotype.

Protein

DNAm ADM was significantly associated with the levels of 16/92 Olink® proteins, 15 of these associations remained significant following adjustment for age 11 IQ (Supplementary File 5b).

DNAm Beta-2 microglobulin

Physical

Age-adjusted DNAm Beta-2 microglobulin (B2M) was positively associated with levels of interleukin-6 (β = 0.16, SE = 0.04, P = 3.0 x 10^{-3}).

Cognitive

Age-adjusted DNAm B2M was not significantly associated with any cognitive phenotype.

Neuroimaging

Age-adjusted DNAm B2M was negatively associated with the ratios of grey matter volume:ICV (β = -0.16, SE = 0.04, P = 7.0 x 10^{-3}) and brain volume:ICV (β = -0.16, SE = 0.04, P = 7.0 x 10^{-3}).

Protein

DNAm B2M was significantly associated with the levels of 6/92 Olink® proteins, 5 of these associations remained significant following adjustment for age 11 IQ (SIGLEC1, MSR1, IL12, SCARB2 and Beta NGF) (Supplementary File 5c).

DNAm Cystatin C

Physical

Age-adjusted DNAm Cystatin C was positively associated with levels of interleukin-6 (β = 0.14, SE = 0.04, P = 5.0 x 10^{-3}) and body mass index (β = 0.14, SE = 0.04, P = 5.0 x 10^{-3}).

Age-adjusted DNAm Cystatin C was negatively associated with levels of low-density lipoprotein cholesterol (β = -0.14, SE = 0.04, P = 5.0 x 10^{-3}), total cholesterol (β = -0.12, SE = 0.04, P = 0.01), iron (β = -0.14, SE = 0.04, P = 0.01) as well as forced vital capacity (β = -0.08, SE = 0.04, P = 0.04). All associations remained significant following controlling for age 11 IQ.

Cognitive

After adjusting for age 11 IQ, age-adjusted DNAm Cystatin C was negatively associated with the g factor (β = -0.09, SE = 0.03, P = 0.04).

Neuroimaging

Age-adjusted DNAm Cystatin C was negatively associated with the ratios of grey matter volume:ICV (β = -0.20, SE = 0.04, P = 8.6 x 10^{-5}), brain volume:ICV (β = -0.20, SE = 0.04, P = 2.5 x 10^{-4}) and white matter volume:ICV (β = -0.14, SE = 0.04, P = 0.02). All associations were significant after correcting for age 11 IQ.

Protein

DNAm Cystatin C was significantly associated with the levels of 3/92 Olink® proteins, all of which remained significant following adjustment for age 11 IQ (G CSF, SIGLEC9 and MSR1) (Supplementary File 5d).

DNAm Growth Differentiation Factor 15

Physical

Age-adjusted DNAm Growth Differentiation Factor 15 (GDF15) was not associated with any physical phenotype.

Cognitive

Age-adjusted DNAm GDF15 was not associated with any cognitive phenotype.

Neuroimaging

Age-adjusted DNAm GDF15 was not associated with any neuroimaging phenotype.

Protein

DNAm GDF15 was not significantly associated with the levels of any Olink® protein (Supplementary File 5e).

DNAm Leptin

Physical

Age-adjusted DNAm Leptin was positively associated with levels of interleukin-6 (β = 0.25, SE = 0.07, P = 0.01) and body mass index (β = 0.24, SE = 0.04, P =0.01). After adjusting for age 11 IQ, the association between age-adjusted DNAm Leptin and body mass index did not remain significant (β = -0.21, SE = 0.07, P = 0.05; % attenuation: 14.71%).

Age-adjusted DNAm Leptin was negatively associated with grip strength in the left hand (β = -0.13, SE = 0.02, P = 0.04).

Cognitive

Before adjusting for age 11 IQ, age-adjusted DNAm Leptin was negatively associated with the Wechsler test of adult reading (β = -0.20, SE = 0.07, P = 0.04). However, this association was attenuated following adjustment for age 11 IQ (% attenuation: 36.48%).

Neuroimaging

Age-adjusted DNAm Leptin was negatively associated with the ratios of brain volume:ICV (β = -0.30, SE = 0.07, P = 9.0×10^{-3}) and grey matter volume:ICV (β = -0.23, SE = 0.08, P = 0.04). After adjusting for age 11 IQ, the association between DNAm Leptin and grey matter volume:ICV did not remain significant (β = -0.21, SE = 0.08, P = 0.08; % attenuation: 9.21%)

Protein

DNAm Leptin was significantly associated with the levels of 4/92 Olink® proteins, all of which remained significant following adjustment for age 11 IQ (Beta NGF, SCARB2, EFNA4 and MSR1) (Supplementary File 5f).

DNAm Plasminogen activator inhibitor-1

Physical

Age-adjusted DNAm Plasminogen activator inhibitor-1 (PAI1) was positively associated with levels of triglycerides (β = 0.28, SE = 0.04, P = 1.3 x 10^{-9}), body mass index (β = 0.28, SE = 0.04, P = 1.5 x 10^{-9}), levels of interleukin-6 (β = 0.27, SE = 0.04, P = 2.1 x 10^{-9}), weight (β = 0.21, SE = 0.04, P = 8.2 x 10^{-7}) and levels of C-reactive protein (β = 0.19, SE = 0.04, P = 6.4 x 10^{-5}).

Age-adjusted DNAm PAI1 was negatively associated with three measures of lung function: forced viral capacity volume (β = -0.19, SE = 0.03, P = 7.6 x 10^{-9}), forced expiratory volume (β = -0.18, SE = 0.03, P = 1.4 x 10^{-7}), peak expiratory flow (β = -0.11, SE = 0.03, P = 0.01) as well as levels of low-density lipoprotein cholesterol (β = -0.21, SE = 0.04, P = 3.3 x 10^{-6}), total cholesterol (β = -0.14, SE = 0.04, P = 3.0 x 10^{-3}), high-density lipoprotein cholesterol (β = -0.12, SE = 0.04, P = 0.02) and grip strength in the left hand (β = -0.07, SE = 0.03, P = 0.03). Only the association between age-adjusted DNAm PAI1 and high-density lipoprotein cholesterol did not remain significant after adjusting for age 11 IQ.

Cognitive

After adjusting for age 11 IQ, age-adjusted DNAm PAI1 was not associated with any cognitive phenotype.

Neuroimaging

Age-adjusted DNAm PAI1 was negatively associated with the ratios of brain volume:ICV (β = -0.24, SE = 0.04, P = 8.2 x 10^{-7}), grey matter volume:ICV (β = -0.21, SE = 0.05, P = 6.4 x 10^{-5}) and white matter volume:ICV (β = -0.16, SE = 0.04, P = 3.0 x 10^{-3}). After adjusting for age 11 IQ, all associations remained significant.

Protein

DNAm PAI1 was significantly associated with the levels of 21/92 Olink® proteins, 19 of these associations remained significant following adjustment for age 11 IQ. Additionally, a further three

proteins were significantly associated with DNAm PAI1 after adjusting for age 11 IQ (G CSF, KYNU and THY1) (Supplementary File 5g).

DNAm Tissue inhibitor of metalloproteinases 1

Physical

Age-adjusted DNAm Tissue inhibitor of metalloproteinases 1 (TIMP1) was positively associated with levels of C-reactive protein (β = 0.15, SE = 0.04, P = 3.0×10^{-3}), interleukin-6 (β = 0.16, SE = 0.04, P = 3.0×10^{-3}), creatinine (β = 0.11, SE = 0.04, P = 0.02) and body mass index (β = 0.10, SE = 0.04, P = 0.04).

Age-adjusted DNAm TIMP1 was negatively associated with levels of iron (β = -0.17, SE = 0.04, P = 3.0×10^{-3}), forced expiratory volume (β = -0.10, SE = 0.03, P = 9.0×10^{-3}), peak expiratory flow (β = -0.10, SE = 0.03, P = 0.01). All associations remained significant after adjusting for age 11 IQ.

Cognitive

Age-adjusted DNAm TIMP1 was not associated with any cognitive phenotype.

Neuroimaging

Age-adjusted DNAm TIMP1 was not associated with any neuroimaging phenotype.

Protein

DNAm TIMP1 was significantly associated with the levels of 16/92 Olink® proteins, 15 of these associations remained significant following adjustment for age 11 IQ. Additionally, a further two proteins were significantly associated with DNAm TIMP1 after adjusting for age 11 IQ (EFNA4 and LAYN) (Supplementary File 5h).
